# The effects of viewing by scrolling on a small screen on the encoding of objects into visual long-term memory

**DOI:** 10.3389/fpsyg.2023.1191952

**Published:** 2023-08-17

**Authors:** Hayato Sho, Hiromi Morita

**Affiliations:** ^1^Graduate School of Comprehensive Human Sciences, Master’s Program in Informatics, University of Tsukuba, Tsukuba, Ibaraki, Japan; ^2^Institute of Library, Information and Media Science, University of Tsukuba, Tsukuba, Ibaraki, Japan

**Keywords:** visual long term memory, image, object, encoding, efficiency, scrolling, scan, moving image

## Abstract

The perception of an image obtained by scrolling through a small screen can differ from the typical perception of a wide visual field in a stable environment. However, we do not fully understand image perception by scrolling on a small screen based on psychological knowledge of visual perception and cognition of images. This study investigated how screen size limitations and image shifts caused by scrolling affect image encoding in visual long-term memory. Participants explored the stimulus images under three conditions. Under the scrolling condition, they explored the image through a small screen. Under the moving-window condition, they explored the image by moving the screen over a masked image; this is similar to looking through a moving peephole. Under the no-window condition, participants were able to view the entire image simultaneously. Each stimulus comprised 12 objects. After 1 h, the samples were tested for object recognition. Consequently, the memory retention rate was higher in the scrolling and moving-window conditions than in the no-window condition, and no difference was observed between the scrolling and moving-window conditions. The time required by participants to explore the stimulus was shorter under the no-window condition. Thus, encoding efficiency (i.e., the rate of encoding information into memory in a unit of time) did not differ among the three conditions. An analysis of the scan trace of the scrolling and window movements in relation to the image revealed differences between the scrolling and moving-window conditions in terms of the scan’s dynamic features. Moreover, a negative correlation was observed between the memory retention rate and image-scrolling speed. We conclude that perceiving images by scrolling on a small screen enables better memory retention than that obtained through whole-image viewing if the viewing time is not limited. We suggest that viewing through a small screen is not necessarily disadvantageous for memory encoding efficiency depending on the presentation mode, and the results show that participants who scrolled fast tended to have worse memory retention. These findings can impact school education and thus suggest that the use of mobile devices in learning has some merit from the viewpoint of cognitive psychology.

## 1. Introduction

When viewing a map or picture on a mobile device, we often enlarge it to look at a visual object in detail, such as a street name or a friend’s face. Most parts of the image extend past the screen; therefore, we must scroll to look at other details, such as the neighboring street name or the face of another friend. Thus, we scroll through the image to successively present the objects on the screen. Henceforth, we define image perception obtained by scrolling on a limited area window as “scrolling perception.” Although it differs from the ordinary direct perception of the surrounding visual scene, scrolling perception has become the usual style of mobile image viewing. It is therefore essential to study and understand scrolling perceptions within a psychological framework. However, as few studies have attempted to understand scrolling perceptions based on psychological knowledge (Loomis et al., 1991; Fujii and Morita, 2020; Milisavljevic et al., 2021), this study aims to investigate perception while scrolling. Viewing images through scrolling has two major features that are noteworthy from the viewpoint of visual cognition: the limitation of the presentation area and the use of image shifting to view the section of interest. Therefore, we explore the effects of these two features on scrolling perception.

Fujii and Morita (2020) conducted a visual search experiment to investigate visual perception of scrolled images. The participants searched for circles in a stimulus image comprising circles and teardrops. Search times were compared between the scrolling condition (where the participants searched through a screen smaller than the stimulus image), the moving-window condition (where they searched by moving the screen, as if moving a peephole over the masked image), and the no-window condition (where they could see the whole image). Consequently, the no-window condition resulted in the shortest search time among the three conditions. Between the two conditions where they searched using a small screen, the search time for the moving-window condition was shorter than that for the scrolling condition. The experimental results showed that a search using a small screen required a longer time than a search in which the entire image was visible. Furthermore, the results showed that searching by moving an image required longer time than searching by moving a screen. Thus, it was concluded that both features of scrolling presentation—limitations of the visible area of the screen and image shifting—affected the efficiency of the visual search. However, to date, no study has examined whether scrolling presentation affects visual cognitive tasks other than visual search. Therefore, we attempt to elucidate the long-term visual memory and investigate whether these two features of scroll presentation affect the encoding of images into memory.

Researchers have studied how we perceive objects and encode them in visual working memory (VWM) and visual long-term memory (VLTM). Treisman et al. proposed a model in which, by focusing attention on an object, the object’s features are perceived to be bound and represented in VWM (Treisman and Gelade, 1980; Treisman and Gormican, 1988; Kahneman et al., 1992). They termed the representation of feature binding “object file.” Luck and Vogel (1997) demonstrated that VWM actively retains up to four objects in the short term. It has been suggested that the longer an object is maintained in VWM, the more likely it is to be transformed into VLTM, although the efficiency of transfer to VLTM can be modulated by various factors such as attention (Hartshorne and Makovski, 2019; Sasin and Fougnie, 2021; Cotton and Ricker, 2022). VLTM has a large capacity. For example, Brady et al. (2008) found that participants who were presented pictures of 2,500 objects for 3 s each later remembered most of them with detailed information.

The effect of perception through a limited-area window on image recognition was studied using a moving-window technique, which limits the visible area around the central visual field by masking the stimuli outside the window. This window followed the participant’s gaze. These experiments reported that visual search and image recognition times were longer when the participant’s visual field was limited to a small area (Saida and Ikeda, 1979; Bertera and Rayner, 2000; Loschky and McConkie, 2002; Foulsham et al., 2011; Nuthmann, 2013, 2014). In addition, a restricted visible area requires observing the image sequentially. Many studies have shown that the sequential observation mode is inferior to the simultaneous observation mode in visual working memory (VWM) tasks (Allen et al., 2006; Brown and Brockmole, 2010). For example, Allen et al. (2006) presented a sample stimulus consisting of four items for 250 ms in the simultaneous condition in Experiment 5. After a blank of 900 ms, they presented one test item and required the participants to respond if it was the same item as that presented in the sample. In the sequential condition, the four items were sequentially presented for 250 ms each. As a result, the accuracy of recognition of feature conjunction was lower for the sequential condition than for the simultaneous condition. However, some studies have shown that sequential observation results in better immediate recognition test performance (Ihssen et al., 2010; Huebner and Gegenfurtner, 2011). Few studies (Yamamoto and Shelton, 2009) have examined whether the sequential observation mode affects the encoding of objects in the image into visual long-term memory (VLTM).

Although the effect of image shifting during viewing on VLTM has rarely been studied, some studies have focused on the movement of objects and their representations in VWM. According to Kahneman et al., (1992) research on the relationship between perception of continuity of an object and its object file, an object-specific preview effect is observed between an object and a distant preview if apparent motion is perceived between them. This suggests that an object’s feature binding is retained in VWM during movement (Hollingworth and Rasmussen, 2010). Therefore, it is assumed that the bound features are retained in the short term, even if the image is moved by scrolling. In addition, Bharti et al. (2020) compared the accuracy of change detection of item identity when the items changed their location randomly from the study to test displays and when they remained at the same location. Location change decreased the accuracy of change detection in the case of simultaneous presentation of items in the study display. In contrast, it did not affect it in the case of sequential presentation of the items. These results suggest that the encoding of items presented sequentially in VWM is independent of changes in the objects’ location. However, research has not examined whether image shifting through scrolling and the movement of objects beyond the screen and out of sight can affect scrollers’ long-term memory of the objects in the image. Because the relative position between objects is maintained and the participants themselves control the movements, image shifting is not expected to interfere with the encoding of objects into the VLTM.

Therefore, our first research question concerned whether the limitations of the visible area of the screen and image shifting affect long-term visual memory encoding and retention. To answer this question, we conducted a psychological experiment in which participants were presented with images that had many objects arranged on them and were required to view the images by scrolling on a small screen to answer the immediate memory test. After 1 h, the participants were administered unexpected and delayed memory tests. To investigate the effect of limitations on the screen’s visible area, we set a no-window condition where the participants viewed the whole image for comparison. To investigate the effect of image shifting during viewing, we set a moving-window condition where the image was fixed and the window could be moved to scan the image. We predicted that viewing using a small screen would increase encoding time and that the sequential mode of observation would deteriorate memory retention. We predicted that a shift in the absolute location of objects during scrolling would not affect memory encoding.

Our second research question concerns whether a relationship exists between dynamic scan properties and VLTM retention Fujii and Morita (2020) analyzed scan traces while searching through the window. They found that the participants made short and fast scrolls, frequently pausing to search for the scrolling condition. In contrast, they made long and slow window movements and fewer pauses in the moving-window condition. Differences in scan smoothness and continuity are thought to cause differences in the search efficiency between the two conditions. However, it is unclear whether these differences in dynamic features were also observed when the participants explored the image in preparation for an immediate memory test. Endress and Potter demonstrated that repetitive exposure to an object in a rapid, serial visual presentation strengthens the object’s long-term memory trace (Endress and Potter, 2014a). Based on this research, we predicted a relationship between the number of presentations of an object on a screen and the object’s retention rate in VLTM. Furthermore, we performed a multiple regression analysis to investigate whether any dynamic scan properties could explain participants’ VLTM retention rates. If we could find a relationship between the scanning properties of scrolling and memory retention, we could have some indication of the stronger encoding of visual information into long-term memory through scrolling.

It is essential to understand VLTM encoding through scrolling because it facilitates the clarification of the merits and demerits of using information technological devices in learning from the viewpoint of cognitive psychology.

## 2. Materials and methods

This study was approved by the Ethical Committee of the Faculty of Library, Information, and Media Sciences at the University of Tsukuba (no. 21-107) and conducted in accordance with the Code of Ethics and Conduct of the Japanese Psychological Association.

### 2.1. Participants

The study participants were 32 undergraduate and graduate students from the University of Tsukuba (22 men and 10 women aged 22–25 years) with normal or corrected-to-normal vision. All the participants were fully informed about the experiment and provided written informed consent.

### 2.2. Apparatus

The stimuli were presented using a 23-inch display with a touch panel (EIZO DuraVision FDF2382WT, 1920 × 1080px). The experiments were conducted in a room under normal lighting conditions. The selected viewing distance was approximately 45 cm, which differed among the participants because they had to adjust the display’s position and tilt to see and touch it conveniently. Stimulus presentation and response acquisition were controlled using MATLAB and PsychToolbox (Brainard, 1997; Pelli, 1997; Kleiner et al., 2007).

### 2.3. Stimulus

Stimulus images consisted of 700 × 700 px and a white background. They were divided into 4 × 4 cells, of which 12 cells were randomly selected and filled with an object picture. The edges of the images were rimmed with a yellow line that was 6 px in width.

A pool of 1,350 object pictures was randomly divided into three groups—A, B, and C—each of which was used under the three conditions described below. A total of 450 objects in each group were randomly divided into 30 sets, with 15 objects per participant. The 15 objects in each set were randomly divided into groups of 12 and the remaining three objects. Twelve objects (referred to as OLD objects) were placed in 12 cells randomly selected from a stimulus image and presented to the participants. Among the OLD objects, three were randomly selected: one was presented in the immediate memory test when it was an OLD object trial, and the remaining two were presented as OLD objects in the delayed memory test. Among the remaining three objects (referred to as NEW objects), one was presented in the immediate memory test as a NEW object trial, and the remaining two were presented as NEW objects in the delayed memory test.

Object images were sourced from Brodeur et al.’s (2010) image pool. The width of the object image was approximately 163 px. The object image was placed at the center of each cell in the stimulus image or at the center of the display in the memory test.

### 2.4. Procedure

**Figure 1:**
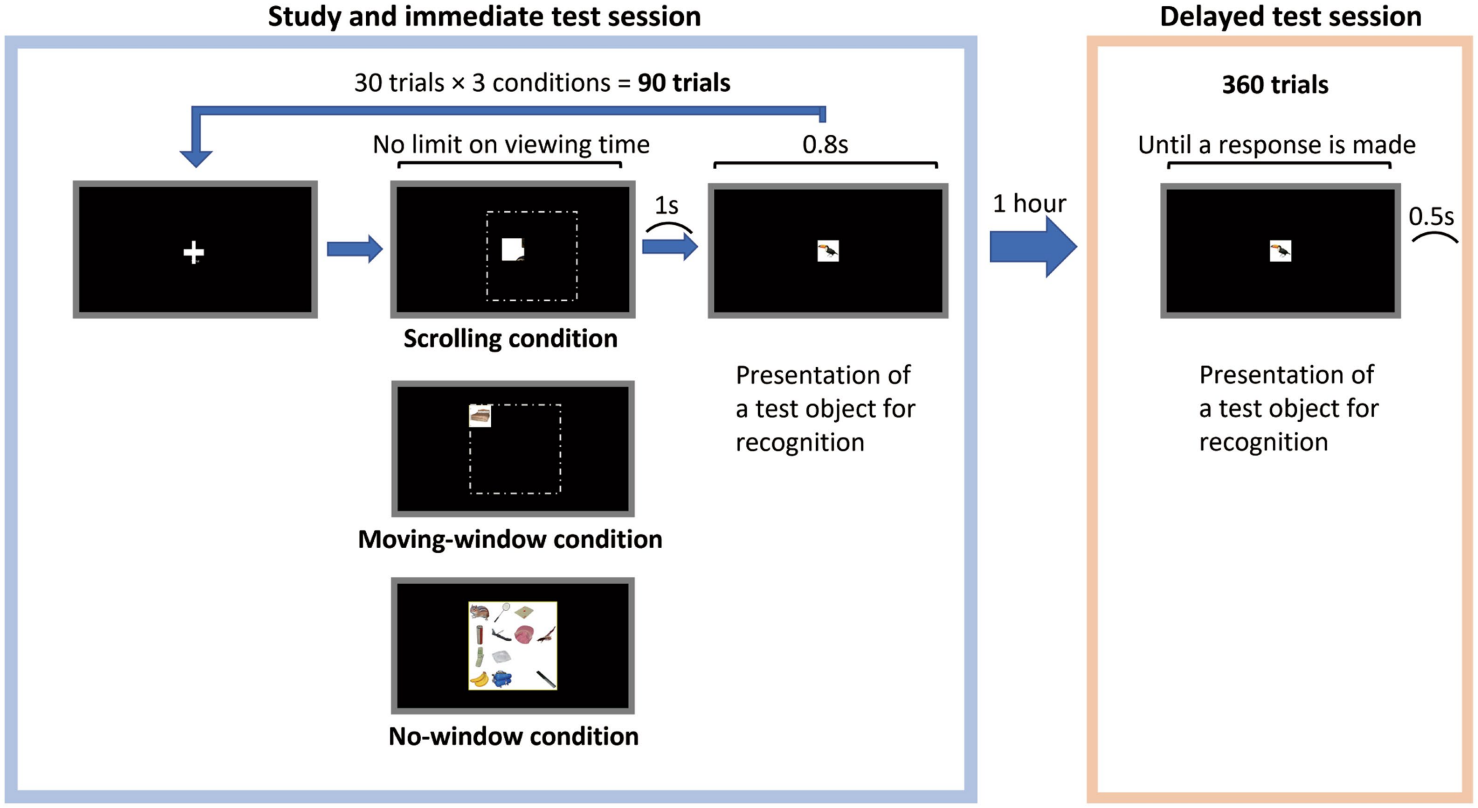
Illustration of the procedure. The dotted lines in the displays for the scrolling and moving-window conditions represent image boundaries. However, these lines have not been shown.

The experiment comprised two sessions. We called the first session the “study and immediate test” and the second session the “delayed test” (see Figure 1).

In the study and immediate test session, when the participants touched the plus mark at the center of the display, the trial started, and an object array image was presented to them. In the scrolling condition, participants were presented with a window through which they could view the image. The window was fixed at the center of the display monitor. The participants could move the image by touching and dragging it into a window to explore it. In the moving-window condition, the participants were presented with a window; however, this time, the image was fixed at the center of the display monitor, and the participants moved the window by touching any area inside it and dragging it to see the entire image. The window was square, and the length of each side was 175 px. It should be noted that scrolling continued even when the participants’ touch was placed outside the window; thus, the length of the dragging movement was not limited to the window width, in comparison with the moving-window condition, where the window followed the participants’ fingers all over the image. Participants were instructed to use the index finger of their dominant hand to touch and drag the screen. In the no-window condition, the entire image was presented to the participants.

However, viewing time was not constrained. Participants who felt that they had fully explored the given image could hit the space key with their non-dominant hand. The display was then blacked out, and after 1 second, the test object was presented at the center of the display for 0.8 s. The participants were required to judge whether this object was the one that they had seen in the stimulus image presented during that trial and to respond by hitting the relevant key with their dominant hand. Half of the trials presented an OLD object, and the remaining half presented a NEW object.

After completing the study and immediate test session, the participants were allowed to take 1 hour of rest, during which time they could do whatever they wanted. They were not informed of what they would have to do in the subsequent session. The delayed test session began when the participants hit the space key. An object was presented at the center of the display, and the participants responded with regard to whether they had seen it in the study and immediate test session using the same process as in the study and immediate test session. When they responded, the trial ended; 0.5 s later, the next trial began. The participants were allowed to rest after every 30 trials. The same number of OLD and NEW object trials was randomly mixed and presented.

### 2.5. Design

In the study and the immediate test session, a block of 30 trials was conducted for each condition. Thus, 1,080 objects were presented to the participants in 90 trials. One object was presented and tested in each trial; thus, a memory test was conducted using 90 objects (45 OLD and 45 NEW objects). Groups of 450 objects (A, B, and C) were used in this order for all the participants; however, the order of the three conditions was counterbalanced. A practice trial was conducted before each block.

In the delayed test session, two OLD objects that were not used in the immediate memory test were randomly selected from the objects presented in each study and immediate test session trial. Therefore, 180 OLD objects and the same number of NEW objects were mixed and presented in random order, and 360 test trials were conducted during the delayed test session.

## 3. Results

We calculated the probability of an object being retained in memory using the formula P = HIT − FA, where HIT represents the hit rate and FA represents the false alarm rate (Endress and Potter, 2014b). By defining efficiency as the amount of information transferred into memory within a fixed time unit (Huebner and Gegenfurtner, 2011), we calculated memory-encoding efficiency (i.e., the number of objects encoded into memory per second) using the formula E = *N* × *P*/*T*, where *N* represents the number of objects presented in a trial and *T* the viewing time.

### 3.1. Viewing time, visual short-term memory retention rate, visual long-term memory retention rate, and visual long-term memory encoding efficiency

**Figure 2:**
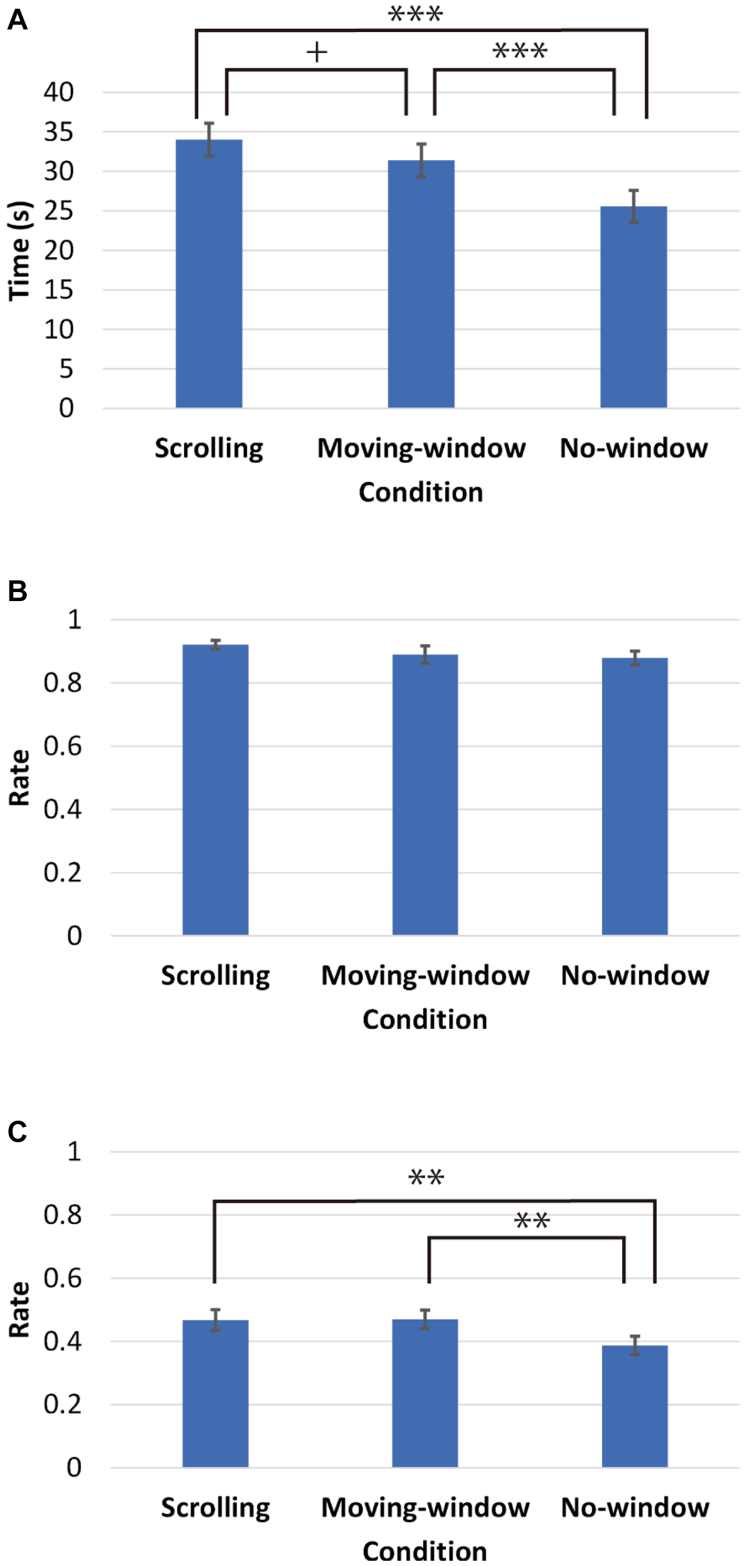
**(A)** Time required to explore an image. **(B,C)** The retention rate measured in the immediate and delayed recognition tests. Error bars represent S.E.

Figure 2A shows the viewing times for the three conditions used in this study. We conducted a one-way repeated-measures analysis of variance (ANOVA) on viewing time with presentation condition as a factor. The main effect of condition was significant: *F*(2, 62) = 24.1, *p* < 0.001, *η*^2^ = 0.438. Multiple comparisons (Bonferroni corrections were applied for this and other multiple corrections in the present study) revealed that viewing time was significantly longer for the scrolling condition than the no-window condition (*p* < 0.001), and for the moving-window condition than the no-window condition (*p* < 0.001). Viewing time was also marginally longer for the scrolling condition than the moving-window condition (*p* = 0.092).

Figure 2B shows the retention rate of visual short-term memory (VSTM) for the three conditions. A one-way repeated-measures ANOVA of the VSTM retention rate did not show any significant effect: *F*(2, 62) = 1.48, *p* = 0.236. The VSTM retention rate was greater than 85% in all conditions.

Figure 2C shows the retention rate of visual long-term memory (VLTM) for the three conditions. A one-way repeated-measures ANOVA conducted on the VLTM retention rate revealed a significant effect of presentation condition: *F*(2, 62) = 7.02, *p* = 0.002, *η*^2^ = 0.185. The retention rate was significantly higher for the scrolling condition than the no-window condition (*p* = 0.008) and higher for the moving-window condition than the no-window condition (*p* = 0.017); however, no difference was observed between the scrolling and moving-window conditions (*p* = 1.00).

A one-way repeated-measures ANOVA conducted on VLTM encoding efficiency showed no significant effect of condition: *F*(2, 62) = 1.82, *p* = 0.171. Multiple comparisons yielded no significant effect between the scrolling and no-window conditions (*p* = 0.312), between the moving-window and no-window conditions (*p* = 1.000). To determine the effectiveness of the condition, we calculated the Bayes factors in favor of the null hypothesis H_0_ versus the opposite hypothesis H_1_. As a result, H_01_ was 1.97 between the scrolling and no-window conditions and 7.29 between the moving-window and no-window conditions. Thus, we detected only anecdotal evidence for H_0_ between the scrolling and no-window conditions and moderate evidence for H_0_ between the moving-window and no-window conditions.

### 3.2. The analysis of dynamic scan properties during scrolling and window moving

For the scrolling and moving-window conditions, we cut the scan traces while viewing the pauses and movements using the following procedures based on Fujii and Morita (2020).

1. We calculated the displacement of a point on the image presented at the center of the window between two consecutive samples. If the displacement was equal to or longer than 2.91 px, the period was categorized as the movement period; if it was shorter than 2.91 px, it was categorized as the pause period. Because the sampling rate was 60 Hz, this critical distance corresponded to a velocity of 175 px/s (5.90 deg./s), that is, the velocity at which the image traveled the width of the window per second.
2. We temporarily categorized the series of consecutive movement periods as one movement and the series of consecutive pause periods as one pause.
3. We then calculated the displacement distance of the window center in relation to the image between a movement’s first and last points. If this distance was shorter than a quarter of the window’s width, 43.75 px, we changed the series of movement periods into pause periods because we considered the series not as a movement but as an adjustment of the image’s location to produce a better view of an object.
4. We then calculated the duration of each pause; if it was shorter than 200 ms, we changed the series of pause periods to movement periods. This is because the usual eye fixation duration is larger than approximately 200 ms and the duration of most fixations in scene perception is approximately 330 ms (Rayner, 1998). We postulate that pauses shorter than 200 ms in duration were made not to perceive the static image but were instead inserted because of the finger’s temporal release.

**Figure 3:**
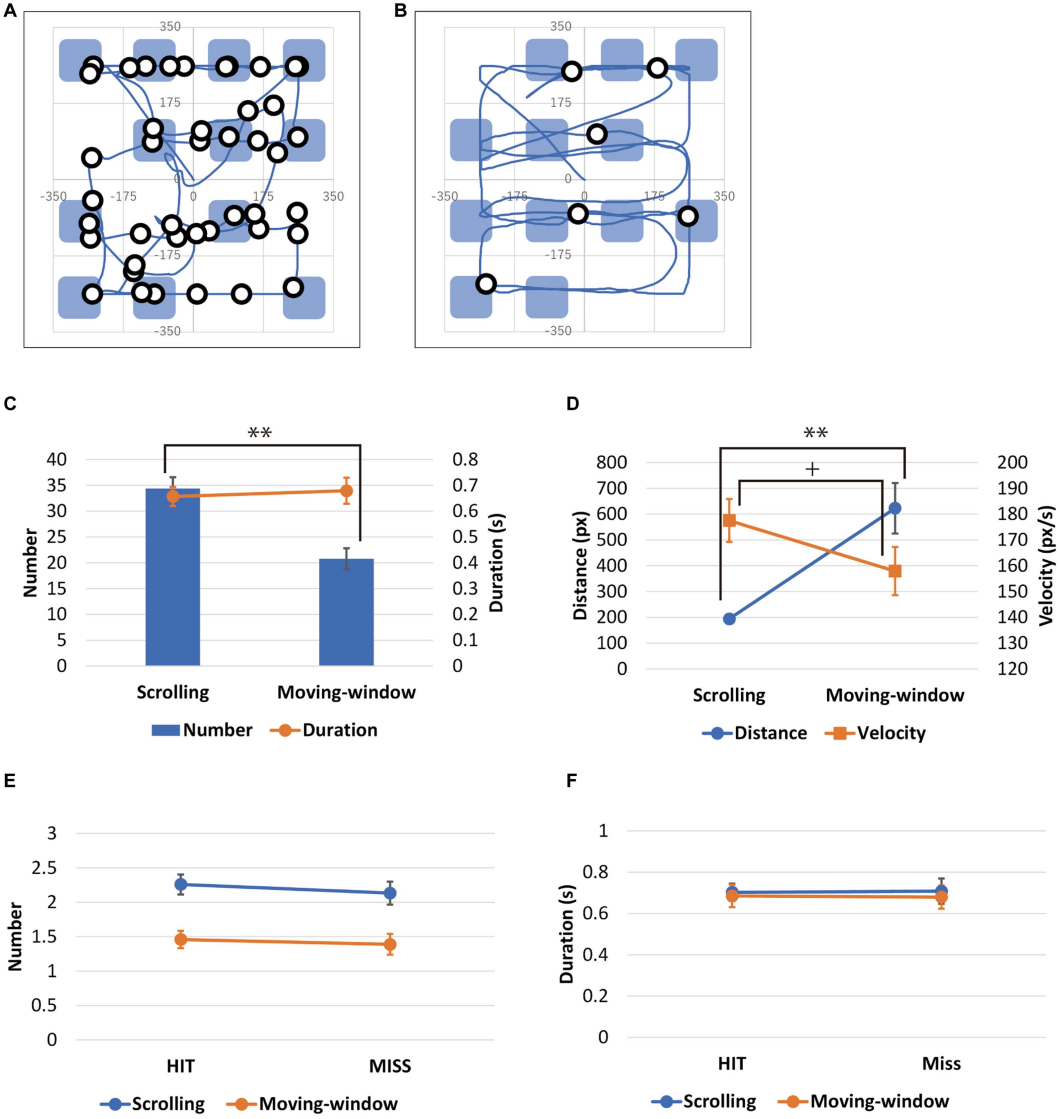
**(A,B)** Scan traces of an experimental participant in the scrolling and moving-window conditions. The lines represent movements, and the small circles represent pauses. The patches represent 12 cells occupied by objects. **(C)** Number of pauses during viewing in a trial and the average pause duration. **(D)** The average distance and velocity of movements. **(E,F)** Number of pauses on the cell of the object that would be presented as a target in the delayed memory test in which the participant hit **(E)** and missed **(F)**. Error bars represent S.E.

Figures 3A,B show the scan traces of one participant in the scrolling and moving-window conditions, categorized into pauses and movements. Participants who exceeded the performance average were excluded from the analysis. This included one participant for whom the average distance of movements in the scrolling condition exceeded the average + 3SD and one for whom the average velocity of movements in the moving-window condition exceeded the average + 3SD. Data from the remaining 30 participants were analyzed.

Figure 3C shows the number of pauses during viewing in a trial and the average duration of pauses under the scrolling and moving-window conditions. The number of pauses was significantly larger for the scrolling condition than for the moving-window condition: *t*(29) = 9.00, *p* < 0.01, *d* = 1.64; however, no significant difference in pause duration was observed between these conditions: *t*(29) = 0.90, *p* = 0.38.

Figure 3D shows the average distance and velocity of the movements under the scrolling and moving-window conditions. The movements’ average distance was significantly longer in the moving-window condition than in the scrolling condition: *t*(29) = 4.83, *p* < 0.01, *d* = 0.88. In addition, their average velocity was significantly higher for the scrolling condition than the moving-window condition: *t*(29) = 1.91, *p* = 0.07, *d* = 0.35.

### 3.3. The comparison of the number of pauses on the HIT and MISS objects

Figures 3E,F show the number and average duration of pauses when points on the presented image at the center of the window were located in the cell of an object that was presented as an OLD object in the delayed memory test. These pauses were divided into pauses on the HIT and MISS objects, depending on whether the participants’ responses to the delayed memory test were correct or incorrect.

We found no significant within-participant difference between the number of pauses for the HIT and MISS objects in the scrolling and moving-window conditions: *t*(29) = 1.43, *p* = 0.16, *t*(29) = 1.16, *p* = 0.26. Furthermore, we observed no within-participant difference between the average pause durations for the HIT and MISS objects in either condition: *t*(29) = −0.15, *p* = 0.88; *t*(29) = 0.16, *p* = 0.87.

The pause durations on both objects were around 700 ms in both conditions.

### 3.4. The relation between VLTM retention rate and dynamic scan properties

The relationship between the VLTM retention rate (dependent variable) and the number of pauses, pause duration, movement velocity, and movement distance (independent variables) was analyzed using multiple regression analysis. Table 1 shows the correlations among the variables. Table 2 presents the results of multiple regression analysis of the scrolling condition. The adjusted *R*^2^ was 0.407, and the model was significant: *F*(4, 25) = 5.971, *p* = 0.002. The velocity of the movement negatively explained the VLTM retention rate (*β* = −0.459, *t* = −2.386, *p* = 0.025). None of the other independent variables had significant β -coefficients.

**Table 1:** The correlations between the retention rate in the delayed memory test and the number of pauses, the duration of the pause, and the distance and velocity of the movement for the scrolling condition.

	Retention rate	Number of pauses	Duration of a pause	Distance of a movement	Velocity of a movement
Retention rate	1	0.25	0.556	−0.261	−0.621
Number of pauses		1	0.004	−0.655	0.016
Duration of a pause			1	−0.121	−0.668
Distance of a movement				1	0.085
Velocity of a movement					1

**Table 2:** Results of the multiple regression analyses for the scrolling condition.

	Standardized *β*	*t*	*p*-values
Constant		1.954	0.062
Number of pauses	0.228	1.199	0.242
Duration of a pause	0.243	1.259	0.220
Distance of a movement	−0.043	−0.226	0.823
Velocity of a movement	−0.459	−2.386	0.025

## 4. Discussion

The first research question aimed to investigate the effects of the features of scrolling presentation using a small screen. Thus, we examined the limitations posed by presentation area and image shifting on the VLTM retention rate and memory-encoding efficiency of the objects in a given image.

Consequently, the no-window condition had the shortest viewing time, which was shorter for the moving-window condition than for the scrolling condition. The immediate memory retention rate was greater than 85% under all conditions, with no significant differences among them. Thus, participants who observed the image spent sufficient time correctly responding to the immediate memory test. The time taken was longer in the two conditions where the screen size was limited, which shows that the screen size limitation increased the time taken to encode the information in the image into the VSTM. Moreover, the time required in the scrolling condition was longer than that in the moving-window condition, indicating that the image shifting that occurred while viewing increased the time required for encoding. This pattern, which we observed for encoding duration among the three conditions, was consistent with the search times observed in a visual search experiment (Fujii and Morita, 2020).

Viewing using a window is expected to require a longer time to encode information because the limitations of the visible area force participants to perform a sequential scan of the image (Saida and Ikeda, 1979). In contrast, the difference between the two conditions in which the visible area was limited demonstrated that the manner in which the sequential scan was conducted affected the time required to encode the information.

The delayed memory test results indicated a lower memory retention rate in the no-window condition than in the scrolling and moving-window conditions; however, no difference was observed between these conditions. The higher performance under the scrolling and moving-window conditions can be attributed to the longer viewing time under these conditions, which enables the transfer of more information to memory. Much rapid serial visual presentation (RSVP) research has shown that the memory encoding of objects or scenes improves when each item receives a longer presentation time or when a longer interval is placed between consecutively presented items (Shaffer and Shiffrin, 1972; Lutz and Scheirer, 1974; Weaver, 1974; Tversky and Sherman, 1975; Potter, 1976).

Thus, the participants spent a longer viewing period scrolling or moving the window, and they retained more objects presented in the image in the VLTM. It should be noted that the participants explored the image so they could answer the immediate memory test, but it is unlikely that they intended to remember the objects in the long term as the delayed test had not been announced in advance. The results of this study are valuable for school education contexts; for example, they demonstrate that if learners use pictures or diagrams on mobile devices with a limited screen area to prepare for the exams that immediately follow, they may take more time, but their long-term consolidation will be better.

To compare memory performance after considering the time spent encoding information into memory, we calculated the memory-encoding efficiency for VLTM, that is, the number of objects encoded into memory per second. No differences were detected among the three conditions. When we calculated Bayes factors to evaluate the evidence for no effect of presentation mode, we found no evidence of any effect between the scrolling and no-window conditions but moderate evidence for no effect between the moving-window and no-window conditions. Therefore, we did not obtain any evidence indicating that scrolling perception degrades long-term memory encoding efficiency compared to the perception of the whole view; moreover, we obtained moderate evidence indicating that moving-window perception does not degrade.

We believe that one of the reasons why memory encoding efficiency did not degrade due to limiting the visible area—at least in the moving-window condition—is that the effect of focusing attention on each item compensated for the cost due to the limited visible area. Participants could focus their attention on each object when they viewed through a small window, resulting in the consolidation of stronger representations in VLTM. Although few studies have examined the effects of focusing attention on encoding objects in VLTM (Williams et al., 2005; Yamamoto and Shelton, 2009; Sasin and Fougnie, 2021), Sasin and Fougnie (2021) show that top-down attention can effectively promote the formation of memory traces in VLTM. It is assumed that, in the present experiment, the participants scrolled or moved the window to show the objects one by one (as shown in Figures 3A,B), which may have enabled them to focus their attention selectively on each object for a certain amount of time and encode it more strongly into the VLTM.

Another possibility is that the participants’ active hand movements to explore the image in the scrolling and moving-window conditions might have enhanced VLTM encoding. Under the no-window condition, too, participants were also able to actively explore the image with their gaze movements. However, eye movements are not always controlled consciously; they are controlled by top-down, memory-based knowledge and bottom-up, stimulus-based information (Henderson, 2003). Therefore, under the no-window condition, it was assumed that participants sometimes moved their gaze almost unconsciously to the salient part of the image. In contrast, under the scrolling and moving-window conditions, the participants might have always consciously moved their hands, which may have enhanced memory encoding. The relationship between active hand movements during scrolling and VLTM encoding is an interesting research subject; however, it is beyond the scope of this study.

While moderate evidence of no difference in encoding efficiency was obtained between the moving-window and no-window conditions, only anecdotal evidence was obtained between the scrolling and no-window conditions. This inconsistency may be because the viewing time for the scrolling condition was slightly longer than that for the moving-window condition. As reported by Fujii and Morita (2020), the scrolling mode is less efficient for searching. Therefore, we speculate that the inconsistency is due to a difference in the efficiency of exploring the image and does not indicate a difference in the memory encoding process. However, future experiments should be conducted with greater discriminability to compare encoding efficiencies among the three conditions in more detail and to test this assumption.

Because no difference was observed in the VLTM retention rate between the scrolling and moving-window conditions, it cannot be said that image shifting during viewing interfered with VLTM retention. This result is consistent with a study that showed that VWM-related task performance is not affected by the objects’ location change during retention when objects are presented sequentially in the study phase (Bharti et al., 2020). However, the present study, focusing on VLTM, differs from the previous study on some points: the objects’ relative position did not change (only their absolute location was shifted by scrolling), and the movement was controlled by the participants themselves. This may also explain why image shifting during scrolling did not interfere with VLTM encoding.

Our second research question aimed to analyze the relevant scan dynamics and investigate the relationship between the VLTM retention rate and dynamic scan features (e.g., number or duration of pauses, velocities, and movement distances).

We categorized the scan traces into pauses and movements to investigate the dynamic properties of the image scanning by participants in the scrolling and moving-window conditions. Consequently, the frequency of pauses was found to be higher and the movement distance shorter in the scrolling condition than in the moving-window condition. This suggests that, when viewing by scrolling, the participants made small movements and frequent pauses, during which they could focus their attention on the object presented on the screen and encode it into their memory. Meanwhile, when viewing by moving the window, the participants made long movements, during which they could focus on the objects so that they made fewer pauses. Because the image was fixed in the moving-window condition, participants could perceive the objects in detail while moving the window. In contrast, as the image moved in the scrolling condition, the participants had to stop scrolling when they perceived the objects in detail. Frequent pauses under the scrolling condition may be related to a longer viewing time. These dynamic scan properties are consistent with those found in a previous visual search experiment (Fujii and Morita, 2020).

Even if a period of exposure to an object as short as the glimpse of one fixation (around 300 ms) is not enough to transfer an object into VLTM, the accumulation of such glimpses increases the possibility of transferring the object into VLTM (Endress and Potter, 2014a). Therefore, we expected recognition performance to be related to the number of pauses. To examine the relationship, we extracted the pauses made on the object to be presented as a target in the delayed memory test. These pauses were categorized into two types based on whether the participants’ answers were correct or incorrect: pauses for HIT objects and for MISS objects. No differences were observed in the number or duration of the two types of pause. This may have been because the average pause duration was as long as 700 ms; thus, the repetition was not always required. However, although this was not significant, the number of pauses made for HIT objects was greater than that made for MISS objects in both conditions (Figure 3E). Therefore, we cannot exclude the possibility that the VLTM retention rate for an object that prompts the participant to pause more frequently is higher. This should be explored in more detail by conducting more detailed experiments.

To identify the types of scrolling that led to better or worse encoding of the image objects into the VLTM, we conducted a multiple regression analysis to examine whether any dynamic scan properties could explain the participants’ memory retention rates for objects. Consequently, a significant negative relationship was identified between movement velocity and memory retention rate. However, no significant relationship was identified between memory retention rate and any other dynamic scan properties, such as the number of pauses, pause duration, and movement distance. The negative relationship between movement velocity and memory retention rate can be explained as follows: a participant who scrolls fast may easily lose the object’s location relative to other objects in the image or the current location where they look in the image; moreover, they do not have enough time to consolidate memory traces because of their momentary movement between objects. Thus, instructing participants who scroll fast and have poor memory retention to scroll slowly could help improve their memory retention rates. Future research could test this assumption from a cognitive-psychological point of view, which could be a strategy for better encoding into VLTM when observing in scrolling mode.

One limitation is that we did not control for participants’ behavior during the delay period between the study and test sessions. Post-learning processes are known to affect VLTM consolidation. For example, encoding new external information or even autobiographical thinking interferes with memory consolidation (Craig et al., 2014), while quiet rest has the least impact (Wamsley, 2022). Based on these studies, it is thought that in the present study, participants might have had different amounts of interference depending on what they did during the delay period. However, the present study was conducted using a between-participants design. Participants underwent a study session of three blocks of different presentation modes; after the delay period, they were tested with old objects selected from the objects presented in these three blocks and new objects, all mixed and randomized. Therefore, any behavior during the delay period would have affected all conditions equally.

## 5. Conclusion

The present study showed that scrolling using a small screen requires more time but enables the retention of more objects in the VLTM. It is suggested that the VLTM encoding efficiency for objects in the stimulus image presented to participants did not differ between the condition of viewing an image through a small screen by moving it and viewing the entire image at once. Viewing multiple objects simultaneously is not always beneficial for memory encoding within a limited period (Yamamoto and Shelton, 2009; Huebner and Gegenfurtner, 2011). Previous studies presented stimuli that were strictly controlled in terms of time and order, whereas the present study allowed participants to observe the image for as long as they deemed sufficient. They were free to look at the objects in any order and as many times as they liked. In other words, the results of the present experiment reflect the participants’ active exploration of the stimulus image through a screen with a limited area.

The present study defined some features of viewing by scrolling through a screen with a limited area, examined the effects of these features on memory encoding, and analyzed scan traces. The relationship between memory retention and scan dynamics under such scrolling condition should be explored in more detail to better understand the features of scrolling perceptions.

## Data availability statement

The raw data supporting the conclusions of this article will be made available by the authors, without undue reservation.

## Ethics statement

The studies involving human participants were reviewed and approved by Ethical Committee of the Faculty of Library, Information, and Media Sciences at the University of Tsukuba. The patients/participants provided their written informed consent to participate in this study.

## Author contributions

HS and HM contributed to the study’s conception and design, performed the statistical analyses, and wrote the manuscript. HS conducted the experiments. All authors contributed to the article and approved the submitted version.

## Funding

This study was supported by JSPS KAKENHI (grant number 21K12602).

## Conflict of interest

The authors declare that this study was conducted in the absence of any commercial or financial relationships that could be construed as potential conflicts of interest.

## Publisher’s note

All claims expressed in this article are solely those of the authors and do not necessarily represent those of their affiliated organizations, or those of the publisher, the editors and the reviewers. Any product that may be evaluated in this article, or claim that may be made by its manufacturer, is not guaranteed or endorsed by the publisher.
